# Modern Rotational Radiation Techniques with Volumetric Modulated Arc Therapy or Helical Tomotherapy for Optimal Sparing of the Lung and Heart in Left-Breast Cancer Radiotherapy Plus Regional Nodal Irradiation: A Comparative Dosimetric Analysis

**DOI:** 10.3390/cancers13205043

**Published:** 2021-10-09

**Authors:** Pei-Yu Hou, Chen-Hsi Hsieh, Le-Jung Wu, Chen-Xiong Hsu, Deng-Yu Kuo, Yueh-Feng Lu, Hui-Ju Tien, Hsiu-Wen Hsiao, Pei-Wei Shueng, Shih-Ming Hsu

**Affiliations:** 1 Department of Radiation Oncology, Far Eastern Memorial Hospital, Taipei 220, Taiwan; jcgv03.be07@nycu.edu.tw (P.-Y.H.); chenci28@ym.edu.tw (C.-H.H.); lejung.tw@yahoo.com.tw (L.-J.W.); cxhsu@mail.femh.org.tw (C.-X.H.); dykuo@mail.femh.org.tw (D.-Y.K.); charmeur0601@yahoo.com.tw (Y.-F.L.); catju@mail.femh.org.tw (H.-J.T.); ep0207@mail.femh.org.tw (H.-W.H.); 2 Department of Biomedical Imaging and Radiological Sciences, National Yang Ming Chiao Tung University, Taipei 30010, Taiwan; 3 School of Medicine, National Yang Ming Chiao Tung University, Taipei 30010, Taiwan; 4 Institute of Traditional Medicine, School of Medicine, National Yang Ming Chiao Tung University, Taipei 30010, Taiwan

**Keywords:** breast cancer, volumetric modulated arc therapy, helical tomotherapy, regional nodal irradiation, organs at risk sparing, deep

## Abstract

**Simple Summary:**

For advanced left-breast cancer patients, adjuvant radiotherapy (RT) with regional nodal irradiation (RNI) has been indicated to reduce cancer recurrence and mortality. Modern arc RT techniques, volumetric-modulated arc therapy (VMAT), or helical tomotherapy (HT), can minimize normal organ exposure without compromising disease control. The aim of this study is to identify which arc technique is optimal for patients receiving left-breast RT with RNI, and to explore distinct RNI volumes with or without IMN. A total of 108 eligible patients were enrolled (70 VMAT, 38 HT). VMAT reduced the mean dose and low-dose exposure to the heart, ipsilateral lung, whole lung, contralateral breast, and esophagus compared with HT. The advantage of VMAT for normal organ sparing was distinct when performing RNI with IMN irradiation. To limit normal organ exposure and reduce potential toxicities, VMAT is the optimal technique for patients with left-breast cancer who are undergoing RT with RNI.

**Abstract:**

Background: For advanced breast cancer with lymph node involvement, adjuvant radiotherapy (RT) with regional nodal irradiation (RNI) has been indicated to reduce cancer recurrence and mortality. However, an extensive RT volume is associated with normal organ exposure, which increases the toxicity and affects patient outcomes. Modern arc RT techniques can improve normal organ sparing compared with conventional techniques. The aim of this study was to explore the optimal technique for left-breast RT with RNI. Methods: We retrospectively reviewed patients receiving RT with RNI for left-breast cancer. We used modern arc RT techniques with either volumetric-modulated arc therapy (VMAT) or helical tomotherapy (HT) with a novel block technique, and compared differences in dosimetry parameters between the two groups. Subgroup analysis of RNI with or without internal mammary node (IMN) volume was also performed. Results: A total of 108 eligible patients were enrolled between 2017 and 2020, of whom 70 received VMAT and 38 received HT. The median RT dose was 55 Gy. No significant differences were found regarding the surgery, RT dose, number of fractions, target volume, and RNI volume between the VMAT and HT groups. VMAT reduced the heart mean dose more than HT (3.82 vs. 5.13 Gy, *p* < 0.001), as well as the cardiac parameters of V5–V20, whole-lung mean dose, lung parameters of V5–V20, and contralateral-breast and esophagus mean dose. In the subgroup analysis of RNI with IMNs, the advantage of VMAT persisted in protecting the heart, lung, contralateral breast, and esophagus. HT was beneficial for lowering the thyroid mean dose. For RNI without IMN, VMAT improved the low-dose exposure of the heart and lung, but HT was similar to VMAT in terms of heart, whole-lung, and contralateral-breast mean dose. Conclusions: For patients with left-breast cancer receiving adjuvant RT with RNI, VMAT reduced the exposure dose to the heart, lung, contralateral breast, and esophagus compared with HT. VMAT was superior to HT in terms of normal organ sparing in the patients who underwent RNI with IMN irradiation. Considering the reduction in normal organ exposure and potential toxicity, VMAT is the optimal technique for patients receiving RNI when deep inspiration breath-hold is not available.

## 1. Background

According to a report by the World Health Organization (WHO), breast cancer was the most commonly diagnosed cancer in 2020, with an estimated 2,261,419 new cases (11.7% of all sites). It was the fifth leading cause of cancer mortality in 2020, with an estimated 684,996 new deaths (6.9% of all sites) [1]. Advances in breast cancer treatment strategies, including surgery, chemotherapy, target therapy, anti-hormone therapy, radiotherapy (RT), and novel immunotherapy, have contributed to improved cancer control and reduced mortality. For the management of patients with advanced breast cancer or lymph node involvement, anthracycline-based chemotherapy, RT with regional nodal irradiation (RNI), and anti-human epidermal growth factor receptor 2 (HER-2) target therapy are commonly used for disease control. However, these agents are associated with cardiotoxicity, and an especially increased risk of heart disease in older survivors [2,3,4,5,6]. As breast cancer patients survive for longer, late side effects due to anticancer therapy are a concern, because they can have adverse effects on clinical outcomes and quality of life.

The large EORTC 22922 randomized trial with 15 years of follow-up data confirmed that RNI with internal mammary node (IMN) and medial supraclavicular fossa (SCF) irradiation significantly reduced mortality and any recurrence in patients with stages I–III breast cancer [7]. However, clinicians face a dilemma when using an extensive RT field to optimize treatment, as this will increase the normal organ exposure dose, potentially leading to higher toxicity and morbidity. The heart and lung exposure dose has been associated with an increase in cardiac mortality and lung cancer incidence even with modern RT techniques [8], with a 0.04 excess rate ratio (ERR) per Gray (Gy) whole-heart dose for cardiac mortality more than 10 years after RT. The association between heart exposure dose and cardiovascular disease (CVD) has also been reported to be a concern, especially for left-side breast cancer [9,10,11,12,13]. For secondary lung cancer, Taylor et al. reported a 0.11 ERR per Gy whole-lung dose [8]. Another case control study of more than 20,000 breast cancer patients also reported that the lung cancer rate after breast RT increased linearly with the lung exposure dose at 8.5% per Gy [14].

Therefore, while RT with extensive RNI is effective for locally advanced breast cancer patients, protecting normal organs to minimize exposure without compromising disease control is an important issue. Modern RT techniques used to optimize dose conformity and spare organs at risk (OARs) are helpful. For a complex RT treatment volume, such as RNI including IMN in left-breast cancer, sophisticated arc-based RT, which combines the dosimetric advantages of rotational delivery with the dose painting capabilities of intensity-modulated radiation therapy (IMRT), is a good choice [15]. Helical tomotherapy (HT) and volumetric-modulated arc therapy (VMAT) are common arc-based approaches. Both techniques improve target coverage and dose homogeneity while concurrently decreasing dose exposure to the heart compared with conventional IMRT or field-in-field techniques for patients requiring RNI [16,17,18,19]. In our previous study on HT, we reported the specific design of an innovative block when planning treatment, which could overcome the disadvantage of low-dose spread to large volumes of the lung, heart, and contralateral breast with traditional HT. We showed that it could provide good dose coverage and reduce the dose to OARs compared with IMRT or traditional HT [20,21].

The primary objective of this study was to identify which modern rotational arc technique is optimal to improve OAR sparing and reduce heart and lung doses for patients with left-breast RT and RNI. The second objective was to explore distinct RNI volumes with or without IMN, and identify the best technique for each specific RNI volume.

## 2. Methods

### 2.1. Patient Population

We retrospectively reviewed patients with breast cancer who were receiving adjuvant RT at Far Eastern Memorial Hospital (FEMH) in Taiwan between 2017 and 2020. The inclusion criteria were patients: with pathologically diagnosed left-side breast cancer, who had undergone lumpectomy or mastectomy surgery, received post-operative RT using modern arc techniques with either VMAT or HT, and who planned to receive RNI. The indications of IMN RT were: ≥4 axillary lymph nodes (LNs) involved, clinically detected IMNs, infraclavicular or supraclavicular LN(s), or a high-risk of having 1~3 LNs involved. The decision to irradiate IMNs was made by the investigator. The exclusion criteria were: bilateral breast cancer, irradiation to only the breast or chest wall without comprehensive RNI, or relatively conventional 3D conformal RT (3D-CRT), IMRT, or hybrid RT. All of the included patients were divided into two technique groups: group 1, VMAT; and group 2, HT. RT treatment factors and dosimetry parameters were compared between the two groups. Subgroup analysis of the patients who received RNI with IMNs or RNI without IMNs was also performed. This study was approved by the Human Experimentation Committee of Far Eastern Memorial Hospital (FEMH-109107-F).

### 2.2. RT Treatment Plan

Computed tomography (CT) simulation with a 2.5 mm slice thickness (Discovery CT590 RT, GE Healthcare, Chicago, IL, USA) was performed for RT treatment planning to delineate the target volume and adjacent OARs. The patients were placed in the supine position and allowed to breathe freely during CT simulation. The radiation volumes included the clinical tumor volume (CTV), including the whole breast or chest wall (CW), and RNI with supraclavicular and infraclavicular regions, any part of the axillary bed at risk, and optional IMN. The planning target volume (PTV) was defined as the CTV plus a 5–8 mm margin for setup error. The RT prescription was a conventional dose of 45–50.4 Gy in 25–28 fractions, or a hypofractionated dose of 40–42.5 Gy in 15–16 fractions with a daily fraction to the breast or chest wall and RNI. An additional 10 to 16 Gy boost dose to the tumor bed or surgical scar was allowed. If there were grossly involved or enlarged unoperated LNs, an additional RT boost could be delivered. The equivalent dose in 2 Gy fractions (EQD2) was evaluated with the α/β ratio of 4 Gy for breast cancer.

### 2.3. RT Technique and Dosimetric Evaluation

VMAT or HT was planned with the aim of accomplishing better homogeneity and conformity of target coverage while sparing adjacent normal organs to minimize exposure to the heart, lung, and other OARs. A dose-volume histogram (DVH) was used to evaluate the radiation dose constraints of the target volumes and OARs. When planning treatment, the following criteria were required: at least 100% of the CTV volume was to receive 100% of the prescription dose, at least 95% of the PTV volume was to receive 95–100% of the prescription dose, and the maximal dose to the PTV region should be less than 110% of the prescription dose. The constraints of OARs were a mean lung dose ≤ 20 Gy, V20 ≤ 35%, a mean heart dose ≤ 20 Gy, V25 < 10%, a maximal spinal cord dose < 45 Gy, and as low a dose as possible to the contralateral breast, with the usual requirements for other normal organs. A Pinnacle3 planning system (version 9.8.1, Philips Medical Systems, Madison, WI, USA) was used to plan treatment for VMAT, which was delivered using a linear accelerator machine (Versa HDTM, Elekta, Crawley, West Sussex, UK) with 6 megavoltage photons. A tomotherapy Hi Art Planning system (version 5.1.3, Tomotherapy, Inc., Madison, WI, USA) was used to plan HT, which was delivered using a Tomotherapy^®^ Hi-Art or HD system (Tomotherapy^®^; Accuray Inc., Madison, WI, USA).

Image verification for treatment consistency was performed using X-ray plain film at least weekly. More frequent image guidance or the use of CT was optional to define the anatomy and internal soft tissue position.

### 2.4. Statistical Analysis

The patients’ characteristics and treatment factors were compared between the two groups using independent Student’s *t* tests for continuous variables, and chi-square tests for categorical variables. The dosimetry parameters were analyzed using independent Student’s *t* tests for a larger sample size, and the Mann–Whitney U test for a smaller sample size. Differences in the results were considered statistically significant when the *p* value was less than 0.05. SPSS software version 22.0 (SPSS Inc., Chicago, IL, USA) was used for all statistical analyses.

## 3. Results

### 3.1. Demographics

**Figure 1:**
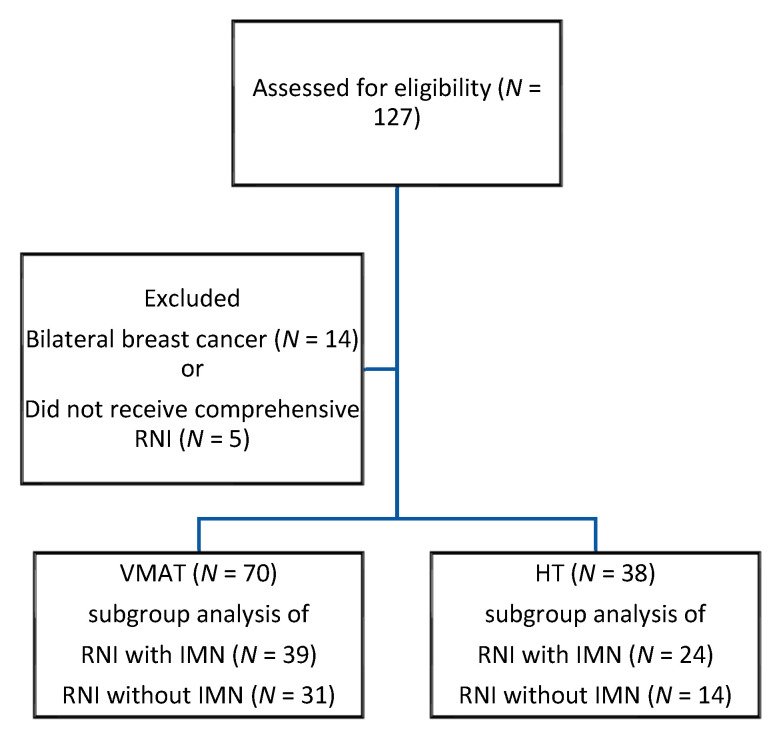
Patient Enrollment. Abbreviations: RNI—regional nodal irradiation; VMAT—volumetric-modulated arc therapy; IMN—internal mammary nodes; HT—helical tomotherapy.

A total of 108 eligible patients with left-breast cancer were enrolled from January 2017 to December 2020. All of them had completed RT with RNI. Seventy patients received the VMAT technique, and the other 38 patients received HT. The process of patient enrollment is shown in Figure 1. The median RT dose was 55 Gy (range 40–74 Gy). No significant differences were found regarding surgery, RT total dose, number of fractions, total dose EQD_2_, conventional or hypofractionation use, CTV, PTV, or RNI volume between the VMAT and HT groups. Details of the patients’ characteristics and RT treatment factors are presented in Table 1.

### 3.2. Comparisons of Dosimetric Outcomes

**Figure 2:**
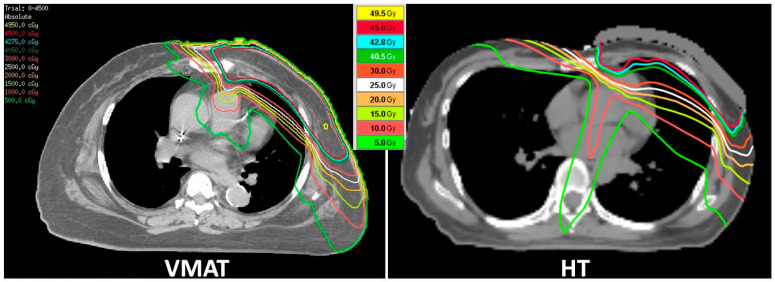
Difference in dose distribution between VMAT and HT. The VMAT technique (**left side**) reduced the normal organ exposure dose, including ipsilateral lung dose V5–V20, heart, contralateral breast and esophagus compared with HT (**right side**).

The heart, ipsilateral-lung, and whole-lung mean doses were 4.27, 10.4, and 5.84 Gy in all eligible patients, respectively. In comparisons of dosimetry parameters between the VMAT and HT groups, normal organ volumes of the heart and lung were similar. The VMAT group had a significantly reduced heart mean dose (3.82 vs. 5.13 Gy, *p <* 0.001) and other cardiac parameters of V5, V10, V15, and V20 (*p <* 0.001, <0.001, <0.001, and 0.02, respectively). The proportion of the heart mean dose ≤ 4 Gy in the VMAT group was 60%, which was better than that in the HT group (26.3%, *p =* 0.002). The VMAT technique had a significantly reduced ipsilateral-lung dose at V5, V10, and V20 (*p <* 0.001, <0.001, and 0.01, respectively), and a trend of a lower left-lung mean dose than HT (9.99 vs. 11.16 Gy, *p =* 0.054). The VMAT group also had a more improved whole-lung mean dose, V5, V10, V20, contralateral-breast mean dose, and esophagus mean dose than the HT group. The dose distribution, delineations of target volumes and OARs, and the DVH of each technique are shown as Figure 2 and Figures S1 and S2. Comparisons of the dosimetry parameters between two techniques for all enrolled patients are listed in Table 2.

We further evaluated specific RNI volumes, considering the most complex target volume with IMN, and a relatively simple volume of RNI without IMN. In the subgroup analysis of RNI with or without IMN, there were no significant differences in the baseline treatment factors, including the RT dose, number of fractions, target volume, or normal organ volumes between the VMAT and HT groups. In the RNI with IMN subgroup, 39 patients received VMAT and 24 patients received HT. The advantage of VMAT persisted in the heart mean dose (4.43 vs. 5.8 Gy, *p =* 0.002), whole-lung mean dose, and low-dose exposure of the heart, ipsilateral lung, and whole lung. The contralateral-breast and esophagus mean doses were also lower in the VMAT group. HT was beneficial in lowering the thyroid mean dose.

In the RNI without IMN subgroup, 31 patients received VMAT and 14 patients received HT. The advantage of VMAT for OAR-sparing was no longer observed in the heart mean dose (3.03 vs. 3.89 Gy, *p =* 0.07), whole-lung mean dose, or contralateral-breast mean dose. However, VMAT was still associated with reducing low-dose exposure of the heart, ipsilateral lung, and whole lung, and the esophagus mean dose. The thyroid mean dose was similar between the two groups if omitting the IMN region. Details are shown in Table 3 and Table 4, respectively. Generally, VMAT was associated with preservation of the heart, ipsilateral and whole lung, contralateral breast, and esophagus in most cases. This advantage was particularly enhanced if IMNs were irradiated.

## 4. Discussion

The indication of distinct RNI volumes is guided by individualized disease status and risk factors. Currently, for patients with pathological N2 disease (≥4 LNs), RNI with IMN is necessary. However, for patients with pathological N1 disease (1–3 LNs) or another high-risk node-negative disease, the criteria for IMN irradiation varies between countries and even between physicians [22]. Because omitting IMNs significantly reduces heart, lung, and contralateral-breast exposure, radiation oncologists evaluate the treatment benefits and toxicity when considering an optional IMN strategy. Nevertheless, few studies have explored modern RT techniques for distinct RNI volumes, and the optimal technique for each treatment volume has yet to be clarified.

For patients with advanced breast cancer with LN metastases, post-operative RT with RNI is shown to reduce mortality and recurrence [7,23]. However, a more extensive RT volume means a greater exposure dose in adjacent normal organs, including the heart and lung, and clinicians have to decide between adequate dose coverage without compromising the treatment effect and RT toxicity. The modern arc RT technique has been shown to achieve superior OAR sparing, dose conformity, and target coverage than conventional 3D-CRT or IMRT for left-breast cancer with RNI [17,24]. When an extensive RT volume is indicated, modern RT is favored in order to protect the normal organs without compromising disease control [16,17,18,19]. In the present study, we focused on modern arc techniques, and compared VMAT and HT for left-breast RT with RNI to clarify which is the preferred technique to reduce OAR exposure and related toxicities. The heart and whole-lung mean doses of 4.27 and 5.84 Gy are comparable with systematic review data on modern RT techniques [8]. VMAT had superior dosimetry parameter profiles for normal organs compared with HT, and was associated with an improvement in heart mean dose, heart V5–V20, lung mean dose, lung V5–V20, and contralateral-breast and esophagus mean dose. This is probably related to the flexibility of the radiation beam angles in VMAT. Because irradiating IMNs increases heart and lung exposure, the IMNs will sometimes be omitted after evaluating the risk of recurrence and treatment toxicity in clinical practice. Thus, we further analyzed the RNI volume with and without IMN involvement to clarify the optimal technique for different indications. Our results showed that the benefit of VMAT was particularly pronounced in the patients who received RNI with IMNs. The advantage of VMAT for OAR sparing diminished with regards to the mean dose to the heart, whole lung, and contralateral breast if the IMNs were not irradiated. Additional IMN irradiation increased the heart mean dose by about 1.5 times compared to not irradiating the IMNs, either with VMAT or HT. The influence of IMN irradiation on the lung was relatively lower. The whole-lung mean dose in the RNI with IMN subgroup was about 1.2 times higher than that in the RNI without IMN subgroup. The difference in ipsilateral-lung mean dose was limited (about 1 Gy), whether or not the IMNs were irradiated.

The issue of heart dose and associated radiation-induced heart disease has gained increasing attention. Heart preservation influences the clinical outcomes and quality of life of cancer survivors, and a proportional increase in the association between major cardiac event rate (myocardial infarction, coronary revascularization, or death from ischemic heart disease) and mean heart dose by 7.4% per Gy with no threshold has been reported. Moreover, the increase continues even into the third decade after breast RT [6]. The dose and volume of the irradiated heart should be as low as possible. However, there is no consensus on a safe threshold. The NSABP-B-51/RTOG1104 study protocol recommends constraints to the heart mean dose of ≤4 Gy and V25 < 10% for left-breast/CW RT [25]. In our patient group receiving RNI, 60% of the patients who received VMAT met the criteria of a mean dose of ≤4 Gy, compared to only 26.3% in the HT group. All patients in both cohorts met the constraint of heart V25 < 10%. RT induces cardiac damage, including vascular endothelium damage, valvular disease, pericarditis, cardiomyocyte damage, conduction dysfunction, and heart failure. The American Society of Clinical Oncology (ASCO) reported that the therapy-related risk factors for developing cardiac dysfunction among breast cancer patients were chest RT ≥ 30 Gy, including the heart in the field, or lower-dose RT (<30 Gy) in combination with lower-dose anthracycline (e.g., doxorubicin <250 mg/m^2^, epirubicin <600 mg/m^2^) [26]. Thus, modern RT techniques have a role in reducing heart exposure, and VMAT is the favored technique to avoid heart impairment in patients receiving left-breast RT with RNI as shown by our results.

With regards to heart exposure, a precise RT field and using at least IMRT or deep-inspiration breath holding (DIBH) is encouraged in the ASCO Clinical Practice Guidelines. A randomized study on left-breast RT with RNI and IMN demonstrated a reduction in cardiac and lung doses in IMRT-DIBH versus 3D-CRT with free breathing [27], and that a lower cardiac dose was associated with clinically meaningful outcomes in the left ventricular ejection fraction and the preservation of cardiac function. This highlights the importance of minimizing the cardiac dose in clinical practice. However, the benefit in OAR sparing is achieved by combining modern IMRT planning techniques with DIBH. To further clarify the advantage of different planning techniques in limiting OAR exposure, it is necessary to disaggregate the impact of RT planning technique from that of breathing status. Hence, in our study, we compared the modern arc planning techniques of VMAT and HT, and both with free breathing while delivering RT. We evaluated the dosimetry profiles of each technique, and avoided confounding from breathing status variables. Although DIBH reduces cardiac dose compared to free breathing in patients receiving left-breast RT with and without RNI [28], not all patients are good candidates for DIBH. The benefit provided by DIBH may be confined to specific populations, such as those with greater lung volume, higher body mass index, postmastectomy, lower quadrant breast tumors, or tumors extending across more than one quadrant [29,30,31,32]. In addition, some patients still have a worse heart and lung dose under DIBH compared with free breathing [30,33]. The effect of DIBH varies individually. Furthermore, around 20–40% of patients are not suitable for or do not benefit from DIBH [30,32,34]. Thus, investigations of optimal techniques should consider situations where DIBH cannot be implement or is not feasible.

The incidence of secondary lung cancer after RT for breast cancer has been reported to increase proportionally with the whole-lung mean dose by 8.5% per Gy [8,14]. VMAT reduced the delivered dose to the lung compared with HT in our study. Although the difference was limited, it is a meaningful result for long-term survivors who are at a higher risk of developing a secondary malignancy. However, with regards to radiation pneumonitis that usually developed in a relatively short period, there is no significant difference between the VMAT and HT groups. The grade 1 pneumonitis were 11.4% vs. 2.6%, and grade 2 pneumonitis were 1.4% vs. 2.6% between the two groups (*p =* 0.27). This has probably been underestimated, especially for the asymptomatic pneumonitis. The grading of pneumonitis is evaluated with the Common Terminology Criteria for Adverse Events version 5.0. Contralateral-breast exposure after RT has been associated with an increased risk of secondary breast cancer. The increased risk of developing cancer has been reported to be dose-dependent, and evident among younger women who undergo RT at <40–45 years of age [35,36]. Our results showed that when adjuvant RT with RNI is used for breast cancer patients, the VMAT technique is favored to spare contralateral breast involvement and avoid the development of a subsequent malignancy. In addition, the benefit is pronounced if the IMNs are irradiated.

The most common thyroid toxicity after RT is hypothyroidism, with an incidence rate of about 20–30% for patients receiving curative RT to the neck region [37,38,39]. Females have a higher incidence of hypothyroidism or thyroid dysfunction after RT. There are currently no definitive OAR constraints for the thyroid. Some studies of normal tissue complications of the thyroid have been conducted in patients with head and neck cancer or Hodgkin’s lymphoma, and these have shown that thyroid V30 or V50 can predict the risk of RT-induced hypothyroidism [40,41]. Data on thyroid tolerance come mainly from patients with other types of cancer. For patients with breast cancer, a prescription dose to the SCF region of 45–50.4 Gy is commonly used, which is comparable with the dose delivered to prophylactic neck lymphatic regions for head and neck cancer. Thus, attention should be paid to thyroid toxicity associated with the RT dose in breast cancer patients, especially for susceptible females. In breast cancer patients receiving RNI, the 3-year hypothyroidism incidence has been three times that compared to patients receiving irradiation to breast alone [42]. The patients in our study used modern arc RT techniques to reduce thyroid exposure and lower toxicity. Our results revealed that in patients indicated for RNI with IMNs, the HT technique can spare the thyroid better than VMAT. However, this superiority diminished if the IMNs were not irradiated.

To the best of our knowledge, this is the largest study of patients with left-breast cancer receiving RNI to analyze the clinical utility of two modern rotational arc techniques. Although conventional 3D-CRT or IMRT can effectively spare the heart, for advanced RNI, and especially when irradiating the IMNs, other OARs including the lung, contralateral breast, and thyroid need to be considered, as well as the heart. In these patients, modern rotational arc techniques are the optimal choice. DIBH has good heart-sparing ability. However, it is a time-consuming procedure that is not suitable for all patients. The VMAT technique can be performed with a linear accelerator, which are commonly used in RT departments and are suitable for nearly every indicated patient. We used whole-heart dosimetry parameters, but not the left anterior descending artery, for evaluation, because delineating the left anterior descending artery is difficult in a CT simulation without contrast, as well as being operator-dependent. Considering the accuracy and reproducibility, whole-heart dose is still standard for DVH constraint evaluation.

There are some limitations to this study. It is a retrospective study performed at a single institution. These results are based on institutional clinical practice experience, and may not represent other clinics. There are fewer patients in the HT group, nearly half of those in the VMAT group, which may have affected the results. The small number of patients in the subgroup analysis is correlated to heterogeneous patient characteristics and dosimetry parameters of RT planning. It will be difficult to obtain more precise results for distinct RNI volumes, either with or without IMN. Further analysis of specific cardiac substructure DVHs that may be predictive factors or surrogates for radiation-induced heart disease, and integration with clinical surveillance tools, such as serum cardiac biomarkers (troponins, natriuretic peptides), echocardiogram, myocardial perfusion imaging, or other cardiac images may be helpful to clarify the relationship between RT exposure and clinical function impairment.

## 5. Conclusions

For left-breast cancer patients receiving adjuvant RT with RNI, the modern VMAT planning technique reduced the mean dose and low-dose exposure to the heart, ipsilateral lung, whole lung, contralateral breast, and esophagus compared with HT. The advantage of VMAT for normal organ sparing was distinct when performing RNI with IMN irradiation. The only distinct benefit of HT was in lowering the thyroid dose if IMNs were involved. To limit OAR exposure and reduce potential toxicities, VMAT is the optimal technique for patients with breast cancer undergoing RT with an extensive irradiation volume, and is recommended for patients not suitable for DIBH.

## Figures and Tables

**Table 1:** Patient characteristics and RT treatment factors.

Characteristics	VMAT (*n* = 70)	HT (*n* = 38)	*p*-Value
Lumpectomy: N (%)	35 (50%)	23 (60.5%)	0.30
Mastectomy: N (%)	35 (50%)	15 (39.5%)	
RT total dose (Gy) (SD) (range)	56.4 (6.6) (40–74)	56.5 (5.4) (45–70)	0.97
Number of fractions (SD) (range)	28.7 (4.6) (15–37)	28.8 (4.1) (16–35)	0.92
RT total dose EQD_2_ (Gy)	57.1	57.8	0.77
Conventional fractionation: N (%) Hypofractionation: N (%)	63 (90%) 7 (10%)	34 (89.5%) 4 (10.5%)	1
CTV (mL) (SD) (range)	481.7 (263.8) (158.5–1314.4)	532.3 (383.5) (150.4–1845.6)	0.42
PTV (mL) (SD) (range)	974.3 (382.1) (402.2–2137.8)	1058.8 (544.2) (388.9–2937.1)	0.35
RT target volume			0.44
Breast_ SCF	21 (30%)	10 (26.3%)	
Breast_ SCF_IMN	14 (20%)	13 (34.2%)	
Chest wall CW_ SCF	10 (14.3%)	4 (10.5%)	
Chest wall CW_ SCF_IMN	25 (35.7%)	11 (28.9%)	
RNI volume			0.45
IMN uninvolved	31 (44.3%)	14 (36.8%)	
IMN involved	39 (55.7%)	24 (63.2%)	

Abbreviations: VMAT—volumetric-modulated arc therapy; HT—helical tomotherapy; RT—radiotherapy; Gy—Gray; SD—standard deviation; EQD_2_—equivalent dose in 2 Gy fractions; CTV—clinical tumor volume; PTV—planning target volume; SCF—supraclavicular fossa; IMN—internal mammary nodes; CW—chest wall; RNI—regional nodal irradiation.

**Table 2:** Comparison of RT treatment factors and dosimetry parameters between VMAT and HT in all eligible patients.

Characteristics	Heart			Left Lung			Whole Lung			Other Normal Organs			
	VMAT (*n* = 70)	HT (*n* = 38)	*p*-Value	VMAT (*n* = 70)	HT (*n* = 38)	*p*-Value	VMAT (*n* = 70)	HT (*n* = 38)	*p*-Value		VMAT (*n* = 70)	HT (*n* = 38)	*p*-Value
Volume (cc)	536.9	505.3	0.16	952.1	933.1	0.69	2192.0	2150.8	0.68	Mean dose (Gy)			
Mean dose (Gy)	3.82	5.13	<0.001	9.99	11.16	0.054	5.57	6.32	0.006	Contralateral breast	2.56	3.39	<0.001
Mean ≤ 4 Gy (%)	42 (60)	10 (26.3)	0.002							Thyroid	21.53	20.34	0.32
V5 (%)	13.96	28.53	<0.001	40.03	52.05	<0.001	20.49	27.56	<0.001	Trachea	11.07	11.61	0.53
V10 (%)	6.37	14.52	<0.001	27.94	37.45	<0.001	13.26	17.70	<0.001	Esophagus	5.67	8.64	<0.001
V15 (%)	4.29	8.10	<0.001	-	-	-	-	-	-				
V20 (%)	3.11	4.66	0.02	18.53	22.23	0.01	8.84	10.10	0.050				
V25 (%)	2.16	2.66	0.30	-	-	-	-	-	-	Maximal dose (Gy)			
V30 (%)	1.46	1.49	0.92	-	-	-	-	-	-	Cord	20.10	20.72	0.55

**Table 3:** Comparison of RT treatment factors and dosimetry parameters between VMAT and HT in patient subgroup of RNI with IMN.

Characteristics	Heart			Left Lung			Whole Lung			Other Normal Organs			
	VMAT (*n* = 39)	HT (*n* = 24)	*p*-Value	VMAT (*n* = 39)	HT (*n* = 24)	*p*-Value	VMAT (*n* = 39)	HT (*n* = 24)	*p*-Value		VMAT (*n* = 39)	HT (*n* = 24)	*p*-Value
Volume (cc)	544.5	510.8	0.30	949.2	907.1	0.73	2199.0	2089.7	0.45	Mean dose (Gy)			
Mean dose (Gy)	4.43	5.80	0.002	10.30	11.23	0.11	6.02	6.82	0.006	Contralateral breast	2.92	3.71	<0.001
V5 (%)	18.31	34.44	<0.001	40.80	54.56	<0.001	22.31	30.48	<0.001	Thyroid	22.08	19.58	0.02
V10 (%)	8.18	17.11	<0.001	28.80	38.93	<0.001	14.36	19.14	<0.001	Trachea	10.93	10.94	0.72
V15 (%)	5.44	9.28	0.004	-	-	-	-	-	-	Esophagus	5.74	8.71	<0.001
V20 (%)	3.82	5.23	0.13	19.21	22.74	0.04	9.69	10.61	0.72				
V25 (%)	2.59	2.89	0.61	-	-	-	-	-	-	Maximal dose (Gy)			
V30 (%)	1.77	1.60	0.45	-	-	-	-	-	-	Cord	20.19	20.17	0.88

**Table 4:** Comparison of RT treatment factors and dosimetry parameters between VMAT and HT in patient subgroup of RNI without IMN.

Characteristics	Heart			Left Lung			Whole Lung			Other Normal Organs			
	VMAT (*n* = 31)	HT (*n* = 14)	*p*-Value	VMAT (*n* = 31)	HT (*n* = 14)	*p*-Value	VMAT (*n* = 31)	HT (*n* = 14)	*p*-Value		VMAT (*n* = 31)	HT (*n* = 14)	*p*-Value
Volume (cc)	527.3	495.3	0.55	955.8	977.7	0.51	2183.2	2255.6	0.62	Mean dose (Gy)			
Mean dose (Gy)	3.05	3.89	0.07	9.61	11.04	0.01	5.02	5.48	0.08	Contralateral breast	2.11	2.84	0.29
V5 (%)	8.48	17.63	0.007	39.07	47.76	0.001	18.19	22.54	0.003	Thyroid	20.80	21.73	0.61
V10 (%)	4.10	9.73	0.004	26.87	34.91	0.001	11.87	15.24	0.001	Trachea	11.25	12.76	0.22
V15 (%)	2.84	5.89	0.03	-	-	-	-	-	-	Esophagus	5.58	8.53	0.002
V20 (%)	2.23	3.62	0.12	17.68	21.36	0.02	7.77	9.24	0.02				
V25 (%)	1.61	2.24	0.14	-	-	-	-	-	-	Maximal dose (Gy)			
V30 (%)	1.06	1.29	0.16	-	-	-	-	-	-	Cord	19.97	21.65	0.13

## Data Availability

The data presented in this study are available on request from the corresponding author. The data are not publicly available due to patients’ privacy and medical ethics.

## Supplementary Materials

The following are available online at www.mdpi.com/article/10.3390/cancers13205043/s1, Figure S1: The delineations of target volumes and OARs, and the dose-volume histogram of VMAT plan, Figure S2: The delineations of target volumes and OARs, and the dose-volume histogram of HT plan.
