# Supplementary material for: Modern Rotational Radiation Techniques with Volumetric Modulated Arc Therapy or Helical Tomotherapy for Optimal Sparing of the Lung and Heart in Left-Breast Cancer Radiotherapy Plus Regional Nodal Irradiation: A Comparative Dosimetric Analysis
Source: Cancers (Basel). 2021 Oct 9;13(20):5043. doi: 10.3390/cancers13205043 (PMC8534109; doi:10.3390/cancers13205043)
Supplement: Supplementary file 1 [file cancers-13-05043-s001.zip › cancers-1373334-supplementary.pdf]

Modern Rotational Radiation Techniques with Volumetric Modulated Arc Therapy or Helical Tomotherapy for Optimal Sparing of the Lung and Heart in Left-Breast Cancer Radiotherapy Plus Regional Nodal Irradiation: A Comparative Dosimetric Analysis

Pei-Yu Hou, Chen-Hsi Hsieh, Le-Jung Wu, Chen-Xiong Hsu, Deng-Yu Kuo, Yueh-Feng Lu, Hui-Ju Tien, Hsiu-Wen Hsiao, Pei-Wei Shueng and Shih-Ming Hsu

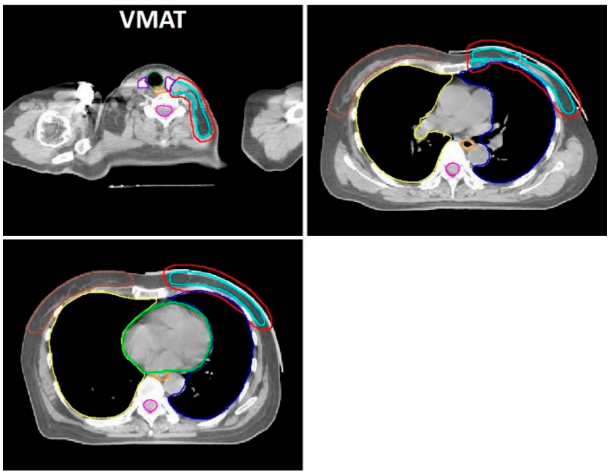

A

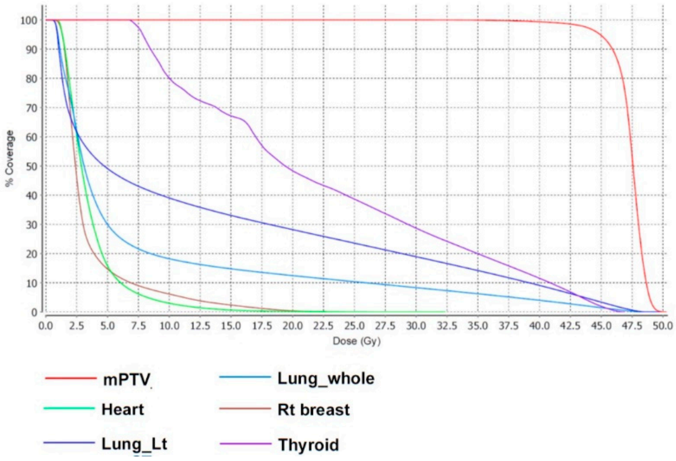

B

Figure S1. The delineations of target volumes and OARs, and the dose-volume histogram of VMAT plan. (**A**) This patient had undergone left modified radical mastectomy, and received adjuvant RT including RNI with IMN using VMAT. The target volumes and OARs were CTV(bright blue), PTV(red), thyroid(purple), trachea(dark green), esophagus(orange), spinal cord(pink), contralateral breast(brown), left lung(blue), right lung(yellow), and heart(bright green). (**B**) The dose-volume histogram (DVH) of VMAT plan. The prescription dose was 45 Gy in 25 fractions to the PTV.

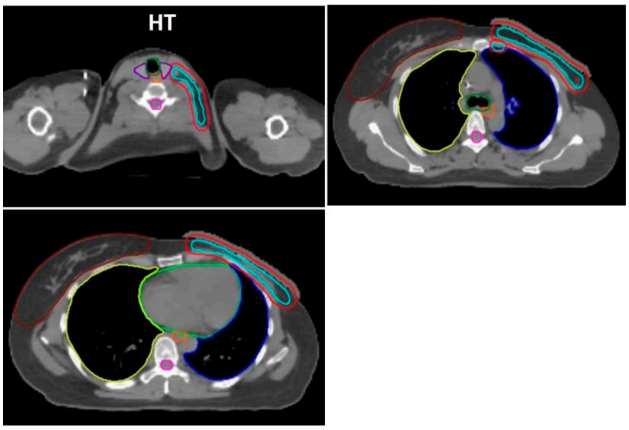

A

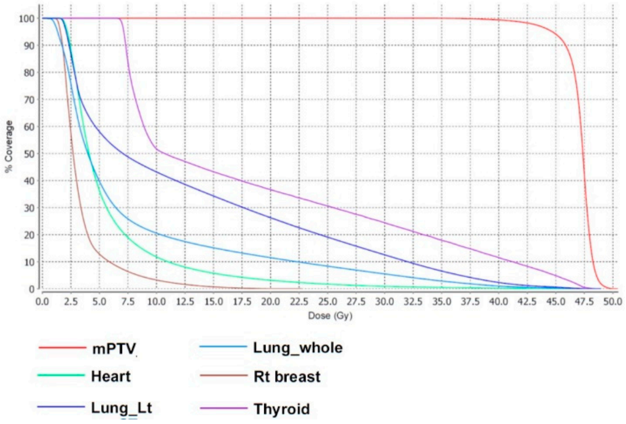

B

Figure S2. The delineations of target volumes and OARs, and the dose-volume histogram of HT plan. (**A**) This patient had undergone left modified radical mastectomy, and received adjuvant RT including RNI with IMN using HT. The target

volumes and OARs were CTV(bright blue), PTV(red), thyroid(purple), trachea(dark green), esophagus(orange), spinal cord(pink), contralateral breast(brown), left lung(blue), right lung(yellow), and heart(bright green). (**B**) The dose-volume histogram (DVH) of HT plan. The prescription dose was 45 Gy in 25 fractions to the PTV.
